# The pleiotropic contribution of genes in dopaminergic and serotonergic pathways to addiction and related behavioral traits

**DOI:** 10.3389/fpsyt.2023.1293663

**Published:** 2023-10-23

**Authors:** Ester Antón-Galindo, Judit Cabana-Domínguez, Bàrbara Torrico, Roser Corominas, Bru Cormand, Noèlia Fernàndez-Castillo

**Affiliations:** ^1^Departament de Genètica, Microbiologia i Estadística, Facultat de Biologia, Universitat de Barcelona, Barcelona, Spain; ^2^Centro de Investigación Biomédica en Red de Enfermedades Raras (CIBERER), Madrid, Spain; ^3^Institut de Biomedicina de la Universitat de Barcelona, Barcelona, Spain; ^4^Institut de Recerca Sant Joan de Déu, Barcelona, Spain; ^5^Psychiatric Genetics Unit, Group of Psychiatry, Mental Health and Addiction, Vall d'Hebron Research Institute (VHIR), Universitat Autònoma de Barcelona, Barcelona, Spain; ^6^Department of Mental Health, Hospital Universitari Vall d'Hebron, Barcelona, Spain; ^7^Centro de Investigación Biomédica en Red de Salud Mental (CIBERSAM), Madrid, Spain

**Keywords:** dopamine, serotonin, addiction, SUD, genetics, aggression, GWAS, pleiotropy

## Abstract

**Introduction:**

Co-occurrence of substance use disorders (SUD) and other behavioral conditions, such as stress-related, aggressive or risk-taking behaviors, in the same individual has been frequently described. As dopamine (DA) and serotonin (5-HT) have been previously identified as key neurotransmitters for some of these phenotypes, we explored the genetic contribution of these pathways to SUD and these comorbid phenotypes in order to better understand the genetic relationship between them.

**Methods:**

We tested the association of 275 dopaminergic genes and 176 serotonergic genes with these phenotypes by performing gene-based, gene-set and transcriptome-wide association studies in 11 genome-wide association studies (GWAS) datasets on SUD and related behaviors.

**Results:**

At the gene-wide level, 68 DA and 27 5-HT genes were found to be associated with at least one GWAS on SUD or related behavior. Among them, six genes had a pleiotropic effect, being associated with at least three phenotypes: *ADH1C*, *ARNTL*, *CHRNA3, HPRT1*, *HTR1B* and *DRD2*. Additionally, we found nominal associations between the DA gene sets and SUD, opioid use disorder, antisocial behavior, irritability and neuroticism, and between the 5-HT-core gene set and neuroticism. Predicted gene expression correlates in brain were also found for 19 DA or 5-HT genes.

**Discussion:**

Our study shows a pleiotropic contribution of dopaminergic and serotonergic genes to addiction and related behaviors such as anxiety, irritability, neuroticism and risk-taking behavior, highlighting a role for DA genes, which could explain, in part, the co-occurrence of these phenotypes.

## 1. Introduction

Addiction is a complex chronic disorder that impacts millions of people around the world (1). Clinically, addiction is now encompassed by the term substance use disorders (SUD) and is characterized in DSM-5 by a core set of behavioral features that can be grouped into impaired control of substance use, impaired social behavior and risky substance use (2).

Transdiagnostic behavioral traits, such as anxiety, irritability, neuroticism, risk-taking behavior or aggressive behavior, have been frequently described in individuals with SUD. Anxiety disorders comprise a heterogeneous group of conditions that often appear as a consequence of stress and previous research has reported an association between SUD and independent anxiety disorders (3, 4). In adults, irritability is regarded as a feature of substance use, disruptive, antisocial and conduct disorder among others, that can be triggered by physiological and environmental stressors (5–7). Neuroticism is a robust personality trait characterized by emotional instability and stress reactivity resulting in the frequent experience of negative emotions that is often associated with a higher risk for developing psychiatric disorders (8). In addition, risk-taking behavior has been closely linked to SUD, aggressive behavior and violence, involving a preference for moderate or high short-term rewards with the potential for a great loss, which is perceived as exciting (9). Finally, recent research has pointed to an association between SUD and aggressive behavior, as these conditions are frequently co-occurring (10, 11). Although all these transdiagnostic behavioral traits have been reported in individuals with SUD, the common genetic and neurobiological factors explaining this co-occurrence are not yet fully understood.

Over the years, research efforts have been made to characterize the neurobiological and psychological underpinnings of SUD and its comorbid behavioral disorders. It is now clear that they are multifactorial disorders, where genetic variation and environmental factors play a role in their development (12–18). Specifically, the critical role of dopamine (DA) and serotonin (5-HT) in addiction processes has been extensively demonstrated over more than 40 years (19–21).

DA circuits are key modulators of behaviors associated with SUD via different mechanisms, and nearly all drugs used by humans acutely increase DA signaling within the striatum (22–24). Additionally, DA signaling is involved in several processes that contribute to the development of addiction, such as reward, learning and motivation (22, 23). Interestingly, DA neurotransmission plays an important role in reward related to aggression, and neurons of medial hypothalamic and mesolimbic circuits modulate this behavior (25). Evidence from behavioral and neuroimaging studies has pointed to a neural imbalance in the reward pathway being involved in retaliatory aggression (26), and research on animal models has described a major contribution of the dopaminergic reward circuitry to appetitive aggression and relapse to aggression seeking (27–29). Moreover, irritability is a core feature of mood disorders, and evidence relates this trait with aberrant striatal responses to DA and low striatal DA levels (5, 7). Finally, several polymorphisms in the *COMT* gene, encoding the enzyme that inactivates catecholamine neurotransmitters, including DA, were previously related to neuroticism and aggressive behavior (30, 31).

The essential involvement of the 5-HT system in both the establishment of drug use-associated behaviors and the transition and maintenance of addiction has been largely studied (20, 32, 33). 5-HT is involved in synaptic plasticity, hedonic tone, motivational and reinforcement processes, learning and memory, all of which are critical processes in the development of addiction (20, 32, 33). Interestingly, selective serotonin reuptake inhibitors are first-line pharmacological treatments for anxiety disorders and have been shown to improve irritability symptomatology in patients with mood disorders (5, 34). Also, 5-HT is a key neurotransmitter with a major role in aggressive behavior largely confirmed by decades of research (35). 5-HT modulates the activity of specific brain areas involved in the control of limbic response, and individuals with increased aggressive behavior have impaired serotonergic functioning in these regions (35).

As described above, dopaminergic and serotonergic neurotransmission have been widely implicated in SUD, and several studies point to their contribution to other related phenotypes including stress-related conditions and aggressive behavior. Decades of research on animal models and candidate-gene association studies have pointed to genes that encode proteins involved in the dopaminergic and serotonergic systems as some of the main genetic contributors to addiction (36, 37). However, the vast majority of the association studies of some of its comorbid phenotypes (16, 38–40), which investigated genetic variants in the core DA and 5-HT genes, were performed in small samples, and the lack of power can explain the contradictory findings or false positive associations (31, 41, 42).

In the present study, we aim to comprehensively assess the genetic contribution of the dopaminergic and serotonergic systems to SUD as well as to other related behavioral traits such as irritability, neuroticism, anxiety, risk-taking behavior and aggressive behavior. These phenotypes co-occur frequently in individuals, and our analyses may contribute to better understand the genetic basis of these comorbidities.

## 2. Materials and methods

### 2.1. DA and 5-HT gene selection

**Figure 1:**
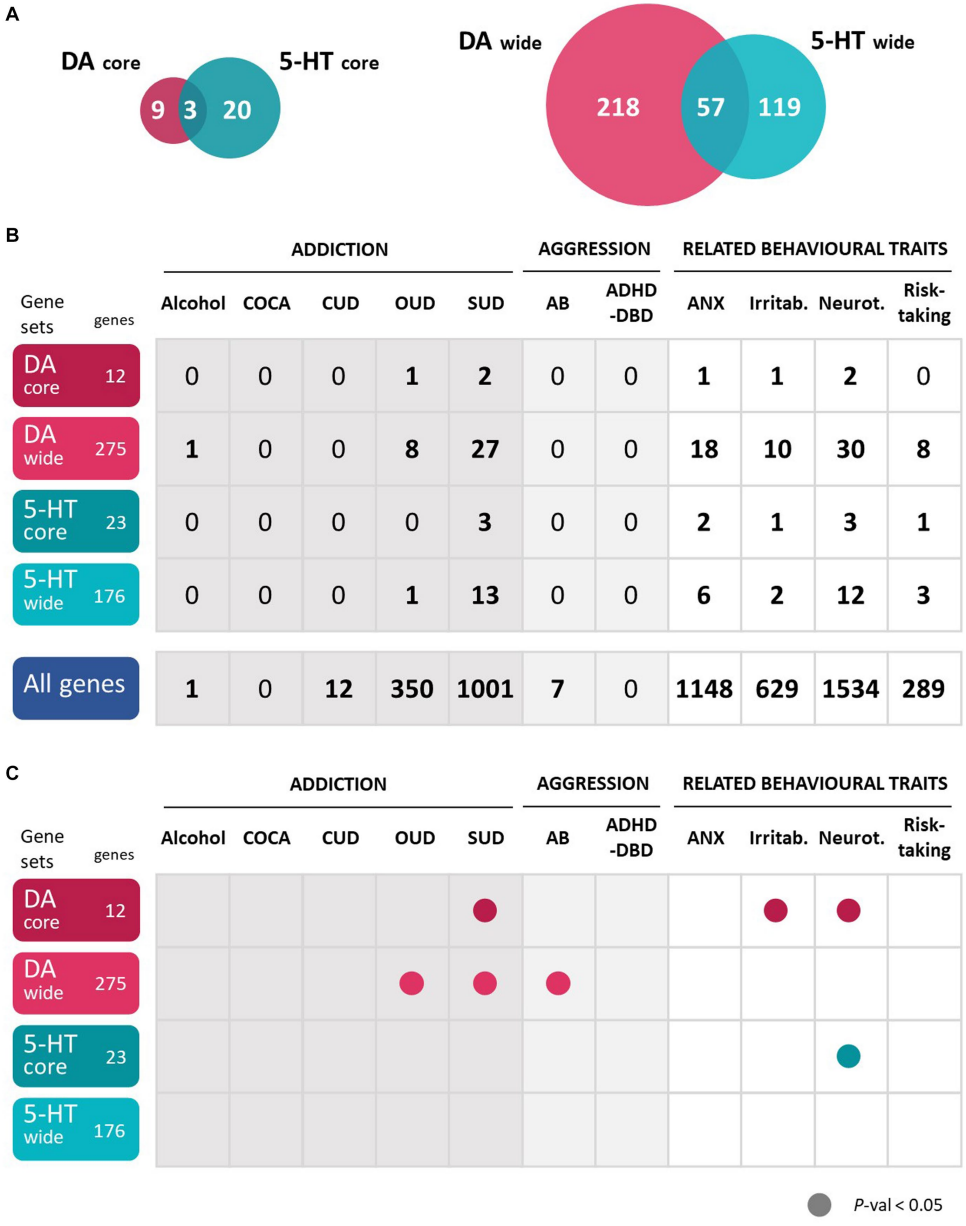
Gene-based and gene-set analyses show that dopaminergic and serotonergic genes are associated with the studied disorders or traits. **(A)** Number of genes belonging to each of the four gene sets analyzed and overlap between them. **(B)** Number of significantly associated genes in the gene-based analyses of 11 disorders or traits. All significant genes overcome a multiple-testing correction of 5% False Discovery Rate, FDR. **(C)** Association of the whole dopamine and serotonin gene sets in the gene-set analyses of 11 disorders or traits. P-val, *p*-value. 5-HT, serotonin; DA, dopamine. In dark gray, addiction disorders; in light gray, aggressive behaviors; in white, related behavioral traits. ADHD-DBD, attention-deficit and hyperactivity disorder comorbid with disruptive behavior disorders; AB, antisocial behavior; Alcohol, alcohol dependence; ANX, anxiety; COCA, cocaine dependence; CUD, cannabis use disorder; Irritab., irritability; Neurot., neuroticism; OUD, opioids use disorder; SUD, substance use disorder; Risk-Taking; risk-taking behavior. All significant genes overcome a multiple-testing correction of 5% False Discovery Rate, FDR.

To comprehensively explore all genes involved in dopaminergic and serotonergic pathways, we used four gene sets previously described by us. Two core gene sets and two wide gene sets were elaborated for dopamine (DA) and serotonin (5-HT) as reported by Cabana-Domínguez et al. (43). The two core gene sets, DA-core with 12 genes and 5-HT-core with 23 genes, were obtained through manual curation and contain exclusively the main genes involved in dopaminergic and serotonergic transmission including neurotransmitter receptors, transporters, and enzymes involved in their anabolism or catabolism. A detailed list of these genes is available in Supplementary Table S1. The two wide gene sets were defined using GO (Gene Ontology Consortium, http://geneontology.org/) and KEGG^1^ datasets [details on this selection can be found in the study from Cabana-Domínguez et al. (43)], obtaining a DA-wide set of 275 genes and a 5-HT-wide set of 176 genes (Supplementary Table S1). The intersection of these lists includes 57 genes that participate in both dopaminergic and serotonergic pathways, three of which belongs to both core sets (*DDC*, *MAOA* and *MAOB*) (Figure 1A; Supplementary Table S1).

Since only some of the summary statistics used included genetic variants located in the X chromosome (*anxiety*, *irritability* and *neuroticism*), 14 genes located in this chromosome could not be tested in most of the datasets: 9 from the DA gene sets (*ATP7A*, *AGTR2*, *FLNA*, *GPR50*, *GRIA3*, *HPRT1*, *MAOA*, *MAOB* and *PPP2R3B*) and 8 from the 5-HT gene sets (*ATP7A*, *ARAF*, *ASMT*, *CACNA1F*, *GPM6B*, *HTR2C*, *MAOA* and *MAOB*), being three of them present in both (*ATP7A*, *MAOA* and *MAOB*).

### 2.2. Data used from GWAS of addiction, aggressive behavior and related traits

In this study, we used publicly available data from different studies of SUD, aggressive behaviors and related traits. We used a total of 19 summary statistics of genome-wide association studies (GWAS) performed in individuals with European ancestry, including 8 datasets of SUD, three of aggressive behavior and 8 of other related behavioral traits. Data were either downloaded from the Psychiatric Genomics Consortium (PGC),^2^ the UKBiobank,^3^ the iPSYCH^4^ or the BroadABC^5^ web pages, or shared by the authors of the GWAS (details in Supplementary Table S2).

### 2.3. Selection of summary statistics based on heritability and variant filtering

The SNP heritability of the 19 GWAS mentioned above (Supplementary Table S2) was estimated using linkage disequilibrium score regression (LDSC) (44) (Supplementary Table S3).^6^ In the case of *cocaine dependence*, *alcohol dependence*, *cannabis dependence*, *cannabis use disorder*, *opioids dependence*, *opioids use disorder*, *ever addicted* phenotype, and *anxiety*, heritability was reported on the liability scale considering the sample and population prevalence of each of them. For the other GWAS, a liability scale could not be used due to the absence of a population prevalence estimate or the use of a continuous scale to define the traits.

A total of 7 GWAS were discarded with a SNP-based heritability estimates h^2^_SNP_ < 0.05, indicating a low genetic contribution (Supplementary Table S3). In the case of opioids addiction, both summary statistics of *opioids dependence* and *opioids use disorder* showed a heritability higher than 5%, but *opioids use disorder* summary statistics was selected for subsequent analyses given the higher number of individuals included in this study (Supplementary Table S2).

In total, 11 summary statistics from GWAS were selected for subsequent analyses, including 5 studies on SUD [*alcohol dependence* (45), *cannabis use disorder* (CUD) (46), *cocaine dependence* (47), *opioids use disorder* (OUD) (48) and a multivariate analysis of three *substance use disorders* (SUD) (49)], two on aggressive behavior [*antisocial behavior* (AB) (50) and *disruptive behavior disorders comorbid with attention-deficit/hyperactivity disorder* (ADHD-DBD) (51)] and four on related behavioral traits (*anxiety, irritability*, *neuroticism* and *risk taking behavior*) (Table 1).

**Table 1:** Details of the 11 summary statistics selected for the gene-based, gene-set and predicted gene expression analyses.

	Trait or disorder	Paper	Source	Individuals
Addiction	Alcohol dependence	Walters et al. (45)	PGC	11,569 cases + 34,999 controls
	Cannabis use disorder	Johnson et al. (46)	PGC	17,068 cases + 357,219 controls
	Cocaine dependence	Cabana-Domínguez et al. (47)	Authors	2,085 cases + 4,293 controls
	Opioids use disorder	Deak et al. (48)	Authors	15,251 cases + 538,935 controls
	Substance use disorder	Schoeler et al. (49)	Authors	Ntot = 187,062, Alcohol use disorder (*n* = 28,757), cannabis use disorder (*n* = 358,534), nicotine dependence (*n* = 244,890), frequency of cigarette (*n* = 245,876), alcohol use (*n* = 513,208), cannabis use (*n* = 24,798)
Aggression	ADHD comorbid with disruptive behavior disorders	Demontis et al. (51)	PGC	3,802 cases + 31,305 controls
	Antisocial behavior	Tielbeek et al. (50)	Broad ABC	16,400 individuals
Related behavioral traits	Anxiety: worrier/anxious feelings *	–	UKBiobank	199,463 cases + 152,370 controls
	Irritability *	–	UKBiobank	97,000 cases + 250,000 controls
	Neuroticism score *	–	UKBiobank	293,006 individuals
	Risk-taking behavior *	–	UKBiobank	326,000 individuals

ADHD, attention-deficit and hyperactivity disorder. * GWAS datasets that contained information about the X chromosome and the only ones in which the 14 DA/5-HT genes present in this chromosome could be analyzed.

Genetic variants from most of the summary statistics used were filtered out by MAF ≤ 0.01 and info-score for imputation quality ≤0.8. There were three exceptions in which variants with a lower imputation quality could not be filtered out because the specific info-score values were missing: antisocial behavior (info-score > 0.6), risk taking (info-score > 0.4), and SUD.

### 2.4. Gene-based and gene-set analyses

The contribution of common variants in the DA/5-HT-related genes to SUD, aggressive behavior or related behavioral traits was assessed through gene-based and gene-set analyses using the 11 GWAS summary statistics selected (Table 1).

Gene-based association studies were performed on MAGMA v1.10 (52) using the SNP-wise mean model, with the test statistic being the sum of −log (SNP *p* value) for SNPs located within the transcribed region (defined on NCBI 37.3 gene definitions). The analysis was performed without window around the gene using the 1,000 Genomes Project Phase 3 (European data only) as a reference panel (53). False Discovery Rate (FDR) was used to correct for multiple testing (5% FDR).

Competitive gene-set analyses were performed for the four sets of genes (DA-core, DA-wide, 5-HT-core and 5-HT-wide) using MAGMA to assess their association with the studied phenotypes. Multiple-testing Bonferroni correction was applied considering 44 gene-set tests (*p* < 0.0011).

### 2.5. Effect on brain volumes

To investigate the effect on brain volumes, summary statistics of GWAS meta-analysis in individuals of European ancestry of 7 subcortical volumes (amygdala, caudate nucleus, hippocampus, nucleus accumbens, pallidum, putamen and thalamus) and intra-cranial volume (13,171 individuals) (54), and cortical thickness and brain surface area (23,909 individuals) (55) were downloaded from the ENGIMA web page.^7^ Details on cut-off thresholds of each phenotype can be found in the ENIGMA web page and publications (54, 55). Gene-based and gene-set analyses were performed with MAGMA for each volumetric brain measure as previously described. FDR was used to correct for multiple testing (5% FDR).

### 2.6. Predicted gene expression correlates in each phenotype

We considered all the SNPs located in each DA and 5-HT gene to infer whether the genetically-predicted expression of each DA and 5-HT gene correlates with the GWAS data of the 11 phenotypes of this study (Table 1). These analyses were carried out on MetaXcan [S-PrediXcan (56–58) and S-MultiXcan (59)] using the summary statistics of each disorder or trait. Prediction elastic-net models were downloaded from PredictDB,^8^ which were constructed considering SNPs located within 1 Mb upstream of the transcription start site and 1 Mb downstream of the transcription end site of each gene and were trained with RNA-Seq data of 13 GTEx (release V8) brain regions: amygdala, anterior cingulate cortex BA24, caudate, cerebellar hemisphere, cerebellum, cortex, frontal cortex BA9, hippocampus, hypothalamus, nucleus accumbens, putamen, spinal cord and substancia nigra. S-PrediXcan was used to analyse the genetically determined expression of genes in each of the 13 brain tissues described above for each of the 11 phenotypes previously selected (Table 1): *alcohol dependence* (45), *cannabis use disorder* (46), *cocaine dependence* (47), *opioids use disorder* (48), *substance use disorders* (49), *antisocial behavior* (50), *disruptive behavior disorders comorbid with ADHD* (51), *anxiety, irritability*, *neuroticism*, and *risk taking behavior*. Then, the information across tissues was combined for each phenotype using a multivariate regression with S-MultiXcan, and a multiple-testing FDR correction (5% FDR) was applied for each phenotype considering all the computed genes tested in the analyses.

## 3. Results

### 3.1. DA and 5-HT genes are associated with addiction and related behaviors

We conducted a comprehensive study to explore the contribution of genes involved in dopaminergic and serotonergic pathways to addiction, aggression and related behaviors. Through gene-based analyses we investigated the association of a total of 275 genes in the DA-wide set and 176 genes in the 5-HT-wide set (57 of them included in both pathways) (Figure 1A; Supplementary Table S1) with the 11 selected phenotypes: SUD [*alcohol dependence*, *cocaine dependence*, *cannabis use disorder* (CUD), *opioids use disorder* (OUD) and a multivariate analysis of three substance use disorders (SUD)], aggressive behavior [*antisocial behavior* (AB) and *attention-deficit and hyperactivity disorder comorbid with disruptive behavior disorders* (ADHD-DBD)] and four on related behavioral traits (*risk taking behavior*, *irritability*, *anxiety* and *neuroticism*) (Table 1).

At the gene-wide level, several genes from both the DA-wide and 5-HT-wide sets were found to be significantly associated (overcoming a multiple testing correction of FDR 5%) with 7 of the analyzed phenotypes (Figure 1B). However, most of the SUD or aggression GWAS lacked power, as shown by the limited number of associated genes in total, and we could not identify associated genes in the DA and 5-HT gene sets (Figure 1B).

**Figure 2:**
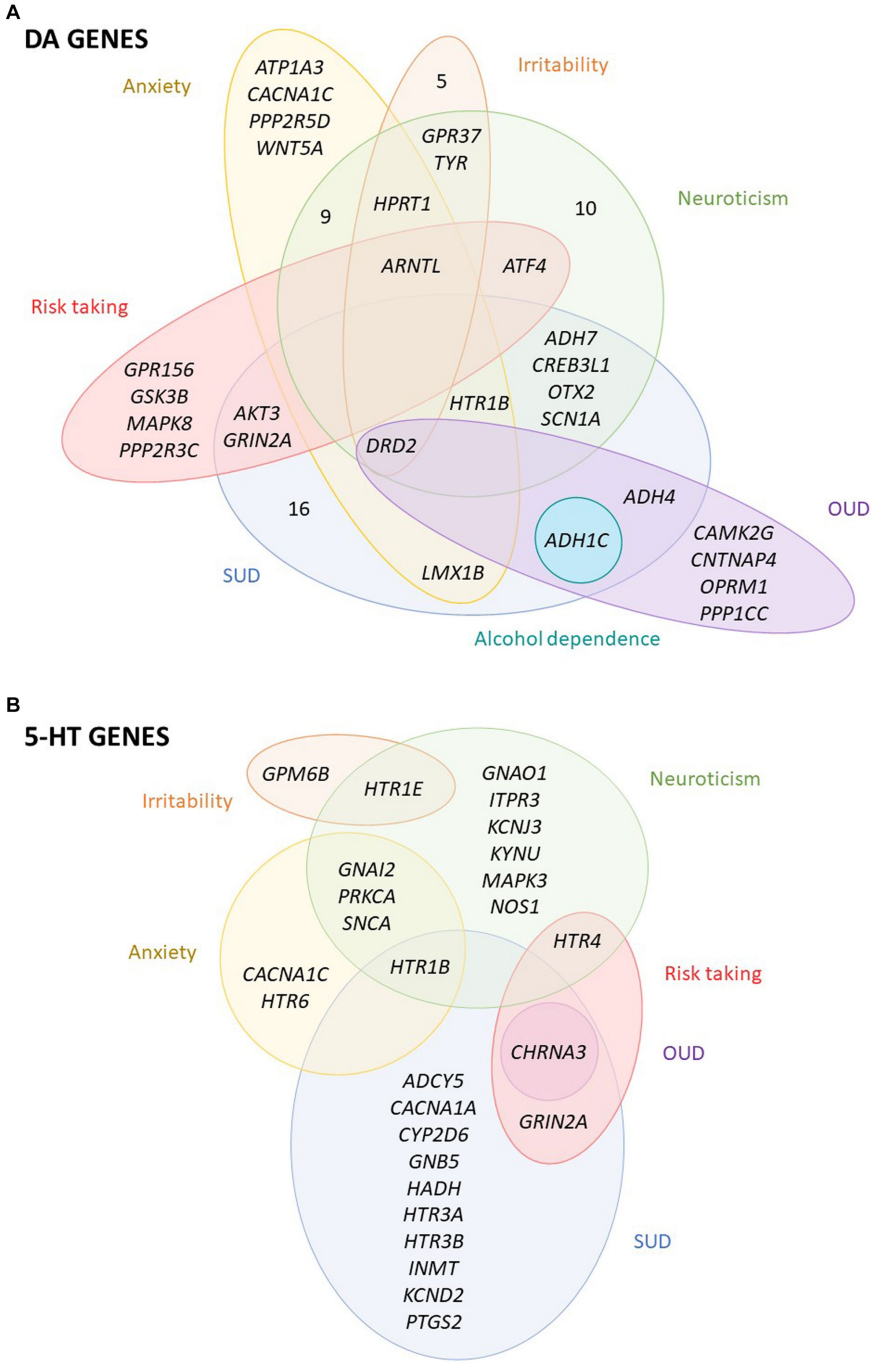
Overlap between the dopaminergic and serotonergic genes associated with the studied disorders or traits. Venn diagrams of the significantly associated **(A)** dopaminergic (DA) and **(B)** serotoninergic (5-HT) genes across the studied disorders or traits. OUD, opioids use disorder; SUD, substance use disorder. All significant genes overcome a multiple-testing correction of 5% False Discovery Rate, FDR.

Among the DA-wide gene set, one gene was found to be significantly associated with alcohol dependence, 8 with OUD, 27 with SUD, 18 with anxiety, 10 with irritability, 30 with neuroticism and 8 with risk taking (Figures 1B, 2A; Supplementary Table S4). Interestingly, five DA genes were found associated with at least three phenotypes, showing a pleiotropic effect: *DRD2*, *ARNTL*, *ADH1C*, *HPRT1* and *HTR1B* (Figure 2A; Supplementary Table S4). In particular, *HPRT1* was associated with the only three phenotypes in which it could be tested: anxiety (*p* = 4.34E-04), irritability (*p* = 2.05E-06) and neuroticisim (*p* = 5.70E-08), since this gene is located in the X chromosome. Three DA core genes were found to be significantly associated with at least one disorder: *DRD2* is associated with OUD (*p* = 6.25E-08), SUD (*p* = 7.74E-13), anxiety (*p* = 3.10E-05), irritability (*p* = 3.41E-05) and neuroticism (*p* = 6.12E-13); *DBH* is associated with SUD (*p* = 3.20E-04); and *DRD3* is associated with neuroticism (*p* = 3.38E-03) (Figure 2A; Supplementary Table S4).

Among the 5-HT-wide genes, one was found to be significantly associated with OUD, 13 with SUD, 6 with anxiety, 2 with irritability, 12 with neuroticism and 3 with risk taking (Figures 1B, 2B; Supplementary Table S5), some of them also present among the DA genes. Interestingly, two 5-HT genes, *HTR1B* and *CHRNA3,* were found significantly associated with three phenotypes (Figure 2B; Supplementary Table S5). Six 5-HT-core genes were found to be significantly associated with at least one disorder: *HTR1B* with SUD (*p* = 3.43E-03), anxiety (*p* = 4.34E-04) and neuroticism (*p* = 9.63E-04), *HTR1E* with irritability (*p* = 1.82E-04) and neuroticism (*p* = 5.89E-05), *HTR4* with neuroticism (*p* = 5.21E-03) and risk taking (*p* = 1.15E-03), *HTR6* with anxiety (*p* = 1.71E-03), and *HTR3A* and *HTR3B* with SUD (*p* = 1.17E-03 and *p* = 1.45E-03, respectively) (Figure 2B; Supplementary Table S5).

Finally, we performed gene-set analyses to assess the contribution of the four gene sets to each of the 11 phenotypes studied (Figure 1C). Interestingly, we found that the DA-wide gene set was nominally associated with antisocial behavior (*p* = 0.022), OUD (*p* = 0.020) and SUD (*p* = 0.017), and that the DA core gene set was nominally associated with SUD (*p* = 0.021), irritability (*p* = 0.006) and neuroticism (*p* = 0.041). On the other hand, the 5-HT core gene set showed exclusively a nominal association with neuroticism (*p* = 0.007) and the 5-HT-wide gene set did not show association with any trait. None of these associations overcame Bonferroni correction.

### 3.2. DA and 5-HT genes associated with volumetric brain changes

We also investigated whether the DA and 5-HT genes were associated with volumetric brain changes by performing a gene-based analysis using data from the ENIGMA consortium. Only the cortical thickness and surface area measures showed significant associations with four DA genes and one 5-HT gene, and no associations were found for the other measures in subcortical regions, probably due to lack of power (Supplementary Figure S1). *AKT3* and *GPR21*, belonging to the DA-wide gene set, and *DDC*, belonging to both DA core and 5-HT core gene sets, were significantly associated with alterations in surface area (*p* = 4.87E-05, *p* = 2.27E-05 and p = 2.27E-04, respectively). In addition, *ATP1A3*, from the DA-wide set, was associated with alterations in cortical thickness (*p* = 5.94E-05). Finally, we performed gene-set analyses and found only a nominal association of the DA-wide gene set with cortical thickness (*p* = 0.015). Interestingly, one of these three genes, *AKT3,* which was associated with alterations in surface area and cortical thickness, was also associated with SUD and risk taking (Supplementary Table S4).

### 3.3. Gene expression correlates in brain regions with addiction, aggression and related behaviors

Finally, we performed a transcriptome-wide association study (TWAs) to explore whether the predicted expression of DA and 5-HT genes correlated with the 11 phenotypes analyzed previously using S-PrediXcan and S-MultiXcan analyses (Supplementary Table S6). Interestingly, the expression of 14 DA, 4 5-HT genes and one gene belonging to both DA and 5-HT gene sets was found to be significantly associated with the phenotype for OUD, SUD, anxiety, irritability or neuroticism (Table 2), and almost all of them were also significantly associated with at least one phenotype in the gene-based analysis. Among them, 7 genes were found to be associated with two traits, the 5-HT gene *CHRNA3* with three traits (OUD, SUD and irritability), and the DA gene *CELSR3* with four traits (OUD, SUD, irritability and neuroticism). We did not find any gene significantly associated with alcohol dependence, cocaine dependence, antisocial behavior or ADHD-DBD, probably due to the lack of statistical power, and no DA and 5-HT genes were found significantly associated with CUD and risk-taking behavior (Supplementary Table S6).

**Table 2:** Genes from the dopaminergic and serotoninergic gene sets significantly associated to at least one disorder or trait in the transcriptome-wide association study of brain tissues performed for 11 disorders or traits.

Genes	Gene sets		Number of tissues^1^	Addiction						Related behavioral traits								
				OUD			SUD			Anxiety			Irritability			Neuroticism		
				*q*-value	Min Z/Max Z	Tissue with best association	*q*-value	Min Z/ Max Z	Tissue with best association	*q*-value	Min Z/Max Z	Tissue with best association	q-value	Min Z/ Max Z	Tissue with best association	q-value	Min Z/ Max Z	Tissue with best association
*ADH1C*	DA		3	**1.42E-02**	−3.26/3.44	Spinal cord	**9.19E-09**	−7.17/3.15	Putamen	–	–	–	–	–	–	–	–	–
*ADH5*	DA		9	4.32E-02	−1.93/3.85	Cerebellar Hemisphere	**3.57E-02**	−1.43/4.08	Spinal cord	–	–	–	–	–	–	–	–	–
*ATF4*	DA		8	–	–	–	–	–	–	–	–	–	–	–	–	**3.29E-04**	−5.13/−1.01	Hippocampus
*ATF6B*	DA		11	–	–	–	–	–	–	**1.54E-04**	3.25/5.58	Frontal Cortex BA9	–	–	–	**8.55E-04**	2.94/5.21	Substantia nigra
*CACNA1C*	DA	5-HT	3	–	–	–	4.97E-02	−2.03/2.75	Cortex	–	–	–	–	–	–	–	–	–
*CELSR3*	DA		1	2.33E-05	5.7/5.7	Amygdala	5.67E-04	4.71/4.71	Amygdala	–	–	–	**7.54E-04**	4.64/4.64	Amygdala	8.76E-04	4.47/4.47	Amygdala
*CHRNA3*		5-HT	4	4.18E-02	0.74/3.68	Cerebellum	1.05E-14	−8.63/−1.57	Cerebellum	–	–	–	–	–	–	–	–	–
*CYP2D6*		5-HT	13	3.67E-02	−0.68/2.49	Cerebellar Hemisphere	1.62E-02	0.69/3.49	Cerebellum	–	–	–	–	–	–	–	–	–
*DRD2*	DA*		3	–	–	–	**1.29E-03**	−4.99/−1.91	Cerebellar Hemisphere	–	–	–	–	–	–	4.76E-03	−4.05/−1.17	Cerebellar Hemisphere
*FLOT1*	DA		8	–	–	–	–	–	–	9.73E-03	−4.75/1.02	Cerebellar Hemisphere	–	–	–	5.93E-03	−5.11/−0.53	Cerebellar Hemisphere
*GABBR1*	DA		8	–	–	–	–	–	–	**5.62E-04**	−1.41/2.65	Cerebellar Hemisphere	–	–	–	**7.89E-04**	−1.71/2.42	Cerebellar Hemisphere
*MAPK3*		5-HT	7	–	–	–	–	–	–	4.68E-02	−0.76/2.9	Caudate	–	–	–	–	–	–
*OPRM1*	DA		1	**1.81E-03**	−4.55/−4.55	Cerebellum	–	–	–	–	–	–	–	–	–	–	–	–
*P2RX1*		5-HT	2	–	–	–	–	–	–	–	–	–	1.34E-02	−3.76/1.16	Nucleus accumbens	–	–	–
*PPP1CC*	DA		2	**2.21E-03**	−4.81/−2.93	Caudate	–	–	–	–	–	–	–	–	–	–	–	–
*PPP2R3A*	DA		2	–	–	–	–	–	–	**2.05E-02**	1.91/3.98	Cerebellum	–	–	–	–	–	–
*TAT*	DA		1	–	–	–	8.93E-03	−3.88/−3.88	Nucleus accumbens	–	–	–	–	–	–	–	–	–
*TIAM1*	DA		4	4.76E-02	−2.78/−1.95	Caudate	–	–	–	–	–	–	–	–	–	–	–	–
*WNT5A*	DA		3	–	–	–	2.20E-02	−0.61/3.48	Cerebellar Hemisphere	–	–	–	–	–	–	–	–	–

5-HT, serotonergic gene set; DA, dopaminergic gene set. * genes present in the DA or 5-HT core gene set. ^1^Number of tissues available for this gene. *q*-value, adjusted value of p after correcting for multiple-testing using a false discovery rate (FDR) approach. Only the genes overcoming a 5% FDR threshold are included in the table and their association to a given disorder is considered significant. Min Z, minimum value of Z-score among the 13 tissues analyzed; Max Z, maximum value of Z-score among the 13 tissues analyzed. In bold, *p*-values of the gene that was also significantly associated to this phenotype in the gene-based analysis.

## 4. Discussion

In this study we have assessed the contribution of common genetic variation of a comprehensive list of genes involved in DA and 5-HT neurotransmission (275 and 176 genes, respectively) to substance use disorders and several related disorders and behavioral traits, including stress-related conditions and aggressive behaviors. Our results show a genetic contribution of both pathways to substance use disorders and several related phenotypes: anxiety, irritability, neuroticism and risk-taking behavior, suggesting a major role for dopamine genes, as pinpointed by the gene-set analyses. We found several genes from both pathways, mostly DA but also 5-HT, associated with the studied phenotypes, and highlighted the contribution of genes that are not core genes but are indirectly involved in DA and 5-HT neurotransmission. Remarkably, 6 of them showed pleiotropic effects, with significant associations with SUD and other related behaviors: *ARNTL*, *ADH1C*, *CHRNA3*, *DRD2*, *HPRT1* and *HTR1B*. Altered expression was predicted for some phenotypes for three of them: *ADH1C*, *CHRNA3* and *DRD2.*

Dopamine gene sets were associated with OUD, SUD and antisocial behavior (DA-wide set), and SUD, irritability, and neuroticism (DA-core set), highlighting a role for dopaminergic genes. These findings are in line with previous results in other psychiatric disorders obtained by our group with the same gene sets, in which we found association of dopaminergic gene sets with ADHD, autism, bipolar disorder, major depressive disorder, Tourette’s syndrome, schizophrenia and a cross-disorder meta-analysis (43). In both studies, most of the genes found associated are not core genes, *DRD2* being the only exception. These results show the relevance of inspecting genes involved in dopamine or serotonin neurotransmission that are not only core genes (receptors, enzymes and transporters).

On the other hand, our gene-set analyses revealed only an association related to the 5-HT genes, specifically between the 5-HT-core gene set and neuroticism. This is in line with previous work showing that thalamic 5-HT transporter binding potentials were associated with neuroticism in both males and females, although with opposite directions (60). Also, polymorphisms in the serotonin transporter were associated with alterations in subnetworks related to cognitive control in women with variable neuroticism scores (30).

When exploring the specific DA and 5-HT genes significantly associated with any of the considered disorders or traits, we found that several of these monoaminergic-related genes were associated with alcohol dependence, OUD and SUD, as well as with the four realated behavioral traits assessed. Unfortunately, in the ADHD-DBD and the antisocial behavior GWAS summary statistics almost no gene-wide association was observed. This prevented us from identifying specific shared candidate genes and disentangling the genetic relationship between addiction and aggression.

Remarkably, the most pleiotropic effect was identified for the *DRD2* gene of the dopaminergic core set, encoding the DA receptor D2, which was associated with OUD, SUD, anxiety, irritability and neuroticism. Indeed, in the GWAS of SUD, the top genetic variant operating through the common liability was located on *DRD2* (49), and *DRD2* showed also a pleiotropic effect in psychiatric disorders being the only gene from the dopaminergic core set associated in the cross-disorder meta-analysis (43). DRD2 has been widely demonstrated to modulate the effects of several drugs of abuse in animal models, especially opiates, alcohol and cocaine (36). Also, systemic injections of Drd2 antagonists were effective reversing the aggressive phenotype in highly aggressive mice (61). Finally, a recent study identified a polymorphism in the *DRD2* gene as related to both anxiety and neuroticism scores in patients with polysubstance use disorder (62). Another gene showing pleiotropic effects is *CHRNA3,* associated with OUD, SUD and risk-taking behavior. This gene encodes an acetylcholine receptor and has been widely associated with nicotine dependence and other SUD (41). The expression of both *CHRNA3* and *DRD2* was predicted to be altered in multiple brain areas in our TWAS, being the cerebellum and the cerebellar hemisphere the tissues with the best association, respectively (Table 1).

Another core gene, *HTR1B*, encoding a serotonin receptor, was associated with SUD, anxiety, and neuroticism. This gene is involved in both DA and 5-HT pathways and associations of genetic variants in it were associated with different SUD but also with aggressive behavior, anger and hostility (31, 63). In the case of *HPRT1*, a gene located on chromosome X, it was associated with the three phenotypes that included this chromosome in the GWAS summary statistics, so it could not be assessed in the majority of the summary statistics. *HPRT1* encodes an important enzyme involved in purine nucleotide exchange that is highly expressed in the central nervous system. In our gene-based analyses, *HPRT1* was associated with anxiety, irritability and neuroticism. Interestingly, alterations in HPRT1 function lead to the Lesch–Nyhan syndrome (LNS), a disorder that presents with nervous systems impairments. In previous studies, a dopamine imbalance was shown for LNS models and *post-mortem* brain from patients, with up to 70–90% decrease of DA levels (64–66). *ARNTL,* encoding a transcription factor, is a clock gene essential for the circadian rhythm that has been previously related to psychiatric disorders (67, 68). In our gene-based analyses, *ARNTL* was associated with the four behavioral traits analyzed: anxiety, irritability, neuroticism and risk-taking behavior. Finally, *ADH1C,* encoding alcohol deshydrogenase 1C, is found to be associated with three addiction disorders: alcohol dependence, OUD and SUD. This can be explained by the inclusion of an alcohol use disorder sample in the SUD summary statistics and the probable co-occurrence of alcohol use disorder in the individuals included in the OUD sample. Also, the expression of *ADH1C* was predicted to be altered in multiple brain areas in the OUD and SUD TWAS analyses.

Certain limitations of this study should be discussed. First, the analysis of the contribution of the DA and 5-HT genes present in the X chromosome could not be properly assessed due to the lack of information from the SNPs situated in this chromosome in several of the GWAS datasets analyzed. This lack of information is not surprising, as only 25% of the GWAS performed nowadays provide results for the X chromosome (69). The X chromosome presents multiple analytical challenges that should be taken into account when analyzing it, and hopefully solved by developing different and specific bioinformatic and statistical analyses in the future (69, 70). In our study, this is the case for 14 genes from our gene sets, whose contribution could not be tested for all the disorders. Especially, we could not properly assess the contribution of *MAOA*, a gene that belongs to both DA and 5-HT gene sets and that had been previously related to aggressive behavior (31, 71). Second, the GWAS data of the four “related traits” analyzed in this study was obtained from the UKBiobank, and there could be some overlapping individuals between the traits. Finally, we could not properly analyze the genetic contribution to aggressive behavior using available GWAS data, due to a lack of power of these studies we did not obtain significant results in the gene-based analysis.

In summary, our results point to a genetic contribution of both DA and 5-HT systems to SUD and several related behavioral traits: anxiety, irritability, neuroticism and risk-taking behavior, highlighting a role for DA neurotransmission, which could explain in part their co-occurrence. More genetic studies on aggressive behavior should be assessed in the future to confirm the contribution of dopaminergic and serotonergic genes to these phenotypes and to better understand the pleiotropic effects of these genes on addiction and other behaviors.

## Data availability statement

The datasets presented in this study can be found in online repositories. The names of the repository/repositories and accession number(s) can be found in the article/Supplementary material.

## Author contributions

EA-G: Formal analysis, Investigation, Methodology, Writing – original draft, Writing – review & editing. JC-D: Conceptualization, Methodology, Supervision, Writing – review & editing. BT: Formal analysis, Writing – review & editing. RC: Supervision, Writing – review & editing. BC: Funding acquisition, Supervision, Writing – review & editing. NF-C: Conceptualization, Funding acquisition, Project administration, Supervision, Writing – original draft, Writing – review & editing.

## Footnotes

^1^ https://www.genome.jp/kegg/pathway.html

^2^ https://pgc.unc.edu/for-researchers/download-results/

^3^ https://www.ukbiobank.ac.uk/

^4^ https://ipsych.dk/en/research/downloads

^5^ http://broadabc.ctglab.nl/summary_statistics

^6^ https://github.com/bulik/ldsc

^7^ https://enigma.ini.usc.edu/

^8^ https://predictdb.org/post/2021/07/21/gtex-v8-models-on-eqtl-and-sqtl/
