# Supplementary material for: The pleiotropic contribution of genes in dopaminergic and serotonergic pathways to addiction and related behavioral traits
Source: Front Psychiatry. 2023 Oct 23;14:1293663. doi: 10.3389/fpsyt.2023.1293663 (PMC10627163; doi:10.3389/fpsyt.2023.1293663)
Supplement: Supplementary file 1 [file Data_Sheet_1.DOCX]

Supplementary Material

# Supplementary Tables

**Supplementary Table 2.** Summary statistics used for the heritability analyses.

|  | Trait or disorder | Paper | Source | Individuals |
| --- | --- | --- | --- | --- |
| **ADDICTION** | Alcohol dependence | (1) | PGC | 11,569 cases + 34,999 controls |
|  | Cannabis dependence | (2) | Authors | 1,822 cases + 5,039 controls |
|  | Cannabis use disorder | (3) | PGC | 17,068 cases + 357,219 controls |
|  | Cocaine dependence | (4) | Authors | 2,085 cases + 4,293 controls |
|  | Ever addicted to any substance or behaviour | - | UKBiobank | 7,000 cases + 11,000 controls |
|  | Opioids dependence | (5) | PGC | 3,272 cases + 25,437 controls |
|  | Opioids use disorder | (6) | Authors | 15,251 cases. 554,186 total individuals |
|  | Substance use disorder | (7) | Authors | Ntot= 187,062, Alcohol use disorder (n=28,757), cannabis use disorder (n=358,534), nicotine dependence (n=244,890), frequency of cigarette (n=245,876), alcohol use (n=513,208), cannabis use (n=24,798) |
| **AGGRESSION** | ADHD comorbid with disruptive behaviour | (8) | PGC | 3,802 cases + 31,305 controls |
|  | Antisocial behaviour | (9) | Broad ABC | 16,400 individuals |
|  | Childhood aggression | (10) | Authors | 87,000 individuals |
| **RELATED BEHAVIOURAL TRAITS** | Anxiety | (11) | iPSYCH | 13,000 cases + 19,000 controls |
|  | Anxiety: mental health problems ever diagnosed by a professional: Anxiety, nerves or generalized anxiety disorder | - | UKBiobank | 16,730 cases + 101,021 controls |
|  | Anxiety: worrier / anxious feelings | - | UKBiobank | 199,463 cases + 152,370 controls |
|  | “Ever been injured or injured someone else through drinking alcohol”: Yes, during the last year | - | UKBiobank | 4,541 cases + 11,3461 controls |
|  | Irritability | - | UKBiobank | 97,000 cases + 250,000 controls |
|  | Neuroticism score | - | UKBiobank | 293,006 individuals |
|  | Risk-taking behaviour | - | UKBiobank | 326,000 individuals |
|  | Risk tolerance | (12) | Authors | 975,353 individuals |

ADHD, attention-deficit and hyperactivity disorder.

**Supplementary Table 3.** Heritability analyses using linkage disequilibrium score regression (LDSC).

|  | Trait or disorder | SNP heritability | Standard error |
| --- | --- | --- | --- |
| ADDICTION | Alcohol dependence | 0.1098 | 0.0206 |
|  | Cannabis dependence | **0.0308** | 0.0488 |
|  | Cannabis use disorder | 0.061 | 0.0051 |
|  | Cocaine dependence | 0.2661 | 0.0515 |
|  | Ever addicted to any substance or behaviour | **0.0077** | 0.002 |
|  | Opioids dependence | 0.1311 | 0.0514 |
|  | Opioids use disorder | 0.238 | 0.0125 |
|  | Substance use disorder | 0.139 | 0.0056 |
| AGGRESSION | ADHD comorbid with disruptive behaviour | 0.1461 | 0.0163 |
|  | Antisocial behaviour | 0.0567 | 0.0263 |
|  | Childhood aggression | **0.0371** | 0.0042 |
| RELATED BEHAVIOURAL TRAITS | Anxiety (iPSYCH) | **0.0192** | 0.0328 |
|  | Anxiety: mental health problems ever diagnosed by a professional: Anxiety, nerves or generalized anxiety disorder | **0.0308** | 0.0036 |
|  | Anxiety: worrier / anxious feelings | 0.098 | 0.005 |
|  | “Ever been injured or injured someone else through drinking alcohol”: "Yes, during the last year" | **0.0009** | 0.0012 |
|  | Irritability | 0.0633 | 0.0034 |
|  | Neuroticism score | 0.0884 | 0.0041 |
|  | Risk-taking behaviour | 0.0549 | 0.0028 |
|  | Risk tolerance | **0.0402** | 0.0015 |

ADHD, attention-deficit and hyperactivity disorder; In bold: SNP heritability lower than 5%.

**Supplementary Table 4**. Dopaminergic genes significantly associated with at least one phenotype in the gene-based analyses of 11 disorders or traits. All genes included in this table overcome a multiple-testing correction of FDR 5%.

| ADDICTION | | | | | |  | RELATED BEHAVIOURAL TRAITS | | | | | | | |
| --- | --- | --- | --- | --- | --- | --- | --- | --- | --- | --- | --- | --- | --- | --- |
| Alcohol dependence | | **OUD** | | **SUD** | |  | **Anxiety** | | **Irritability** | | **Neuroticism** | | **Risk-taking** | |
| Gene name | **p-value** | **Gene name** | **p-value** | **Gene name** | **p-value** |  | **Gene name** | **p-value** | **Gene name** | **p-value** | **Gene name** | **p-value** | **Gene name** | **p-value** |
| *ADH1C* | 1.98E-07 | ***DRD2*** | 6.25E-08 | ***DRD2*** | 7.74E-13 |  | *ATF6B* | 4.62E-10 | *ARNTL* | 7.25E-07 | ***DRD2*** | 6.12E-13 | *AKT3* | 8.09E-08 |
|  |  | *PPP1CC* | 1.02E-06 | *ADH1C* | 2.20E-11 |  | *PPP2R3A* | 5.12E-06 | *HPRT1* | 2.05E-06 | *ARNTL* | 1.72E-09 | *GRIN2A* | 3.42E-05 |
|  |  | *ADH1C* | 5.36E-05 | *CHRNA4* | 2.46E-07 |  | *CACNA1C* | 1.52E-05 | ***DRD2*** | 3.41E-05 | *ATF6B* | 1.88E-09 | *ARNTL* | 1.55E-04 |
|  |  | *CNTNAP4* | 8.23E-05 | *GRIA1* | 4.97E-07 |  | *PRKCA* | 1.77E-05 | *TYR* | 4.63E-05 | *HPRT1* | 5.70E-08 | *MAPK8* | 2.54E-04 |
|  |  | *ADH4* | 3.08E-04 | *OTX2* | 7.46E-07 |  | ***DRD2*** | 3.10E-05 | *GPR37* | 1.86E-04 | *PRKCA* | 4.43E-07 | *GPR156* | 4.05E-04 |
|  |  | *OPRM1* | 6.47E-04 | *ADH1B* | 1.99E-06 |  | *ARNTL* | 6.99E-05 | *TAT* | 2.83E-04 | *KCNJ3* | 1.80E-06 | *ATF4* | 6.54E-04 |
|  |  | *CAMK2G* | 1.52E-03 | *CREB3L1* | 7.18E-06 |  | *PPP2R2B* | 9.68E-05 | *CELSR3* | 1.92E-03 | *CREB3L1* | 1.48E-05 | *GSK3B* | 7.36E-04 |
|  |  |  |  | *GNB5* | 1.85E-05 |  | *HTR1B* | 4.34E-04 | *TYRP1* | 2.31E-03 | *TYR* | 1.74E-05 | *PPP2R3C* | 1.11E-03 |
|  |  |  |  | *ADCY5* | 5.81E-05 |  | *MAPK10* | 6.82E-04 | *DLG4* | 2.39E-03 | *ATF4* | 2.08E-05 |  |  |
|  |  |  |  | *ADH5* | 6.08E-05 |  | *LRRK2* | 8.83E-04 | *GOT1* | 2.82E-03 | *PPP2R2B* | 2.80E-05 |  |  |
|  |  |  |  | *AKT3* | 1.79E-04 |  | *PPP2R5D* | 1.52E-03 |  |  | *NR4A2* | 5.95E-05 |  |  |
|  |  |  |  | *ADH4* | *2.56E-04* |  | *HPRT1* | *1.53E-03* |  |  | *OTX2* | *7.62E-05* |  |  |
|  |  |  |  | *CACNA1A* | 2.93E-04 |  | *PALM* | 1.81E-03 |  |  | *PPP2R3A* | 3.38E-04 |  |  |
|  |  |  |  | ***DBH*** | 3.20E-04 |  | *SNCA* | 1.89E-03 |  |  | *SNCA* | 5.54E-04 |  |  |
|  |  |  |  | *CALML4* | 1.35E-03 |  | *GABBR1* | 2.10E-03 |  |  | *ITPR3* | 6.17E-04 |  |  |
|  |  |  |  | *SCN1A* | 1.59E-03 |  | *ATP1A3* | 2.23E-03 |  |  | *MAPK10* | 7.84E-04 |  |  |
|  |  |  |  | *CHRNA6* | 2.23E-03 |  | *WNT5A* | 4.19E-03 |  |  | *ADH7* | 8.86E-04 |  |  |
|  |  |  |  | *CHRNB2* | 2.92E-03 |  | *LMX1B* | 4.68E-03 |  |  | *HTR1B* | 9.63E-04 |  |  |
|  |  |  |  | *GRIN2A* | 3.03E-03 |  |  |  |  |  | *GABBR1* | 1.98E-03 |  |  |
|  |  |  |  | *ADCY6* | 3.04E-03 |  |  |  |  |  | *GPR37* | 2.42E-03 |  |  |
|  |  |  |  | *LMX1B* | 3.23E-03 |  |  |  |  |  | *GNAO1* | 2.87E-03 |  |  |
|  |  |  |  | *ADH7* | 3.32E-03 |  |  |  |  |  | *GDNF* | 2.91E-03 |  |  |
|  |  |  |  | *HTR1B* | 3.43E-03 |  |  |  |  |  | ***DRD3*** | 3.38E-03 |  |  |
|  |  |  |  | *PPP3CA* | 3.60E-03 |  |  |  |  |  | *PALM* | 3.54E-03 |  |  |
|  |  |  |  | *CREB3* | 3.65E-03 |  |  |  |  |  | *SCN1A* | 4.20E-03 |  |  |
|  |  |  |  | *PTGS2* | 3.95E-03 |  |  |  |  |  | *MAPK9* | 4.31E-03 |  |  |
|  |  |  |  | *CREB3L4* | 4.57E-03 |  |  |  |  |  | *RSPO2* | 4.83E-03 |  |  |
|  |  |  |  |  |  |  |  |  |  |  | *CLOCK* | 5.39E-03 |  |  |
|  |  |  |  |  |  |  |  |  |  |  | *RGS8* | 6.13E-03 |  |  |
|  |  |  |  |  |  |  |  |  |  |  | *LRRK2* | 7.45E-03 |  |  |

In bold, genes belonging to the DA-core gene set. OUD, opioids use disorder; SUD, substance use disorder.

**Supplementary Table 5**. Serotoninergic genes associated with at least one phenotype in the gene-based analyses of 11 disorders or traits. All genes included in this table overcome a multiple-testing correction of FDR 5%.

| ADDICTION | | | |  | RELATED BEHAVIOURAL TRAITS | | | | | | | |
| --- | --- | --- | --- | --- | --- | --- | --- | --- | --- | --- | --- | --- |
| OUD | | **SUD** | |  | **Anxiety** | | **Irritability** | | **Neuroticism** | | **Risk-taking** | |
| Gene name | **p-value** | **Gene name** | **p-value** |  | **Gene name** | **p-value** | **Gene name** | **p-value** | **Gene name** | **p-value** | **Gene name** | **p-value** |
| *CHRNA3* | 2.11E-04 | *CHRNA3* | 1.28E-15 |  | *GNAI2* | 2.56E-07 | ***HTR1E*** | 1.82E-04 | *PRKCA* | 4.43E-07 | *GRIN2A* | 3.42E-05 |
|  |  | *CYP2D6* | 5.17E-06 |  | *CACNA1C* | 1.52E-05 | *GPM6B* | 1.75E-03 | *GNAI2* | 1.23E-06 | *CHRNA3* | 1.06E-03 |
|  |  | *GNB5* | 1.85E-05 |  | *PRKCA* | 1.77E-05 |  |  | *KCNJ3* | 1.80E-06 | ***HTR4*** | 1.15E-03 |
|  |  | *ADCY5* | 5.81E-05 |  | ***HTR1B*** | 4.34E-04 |  |  | *NOS1* | 3.65E-06 |  |  |
|  |  | *KCND2* | 1.57E-04 |  | ***HTR6*** | 1.71E-03 |  |  | ***HTR1E*** | 5.89E-05 |  |  |
|  |  | *CACNA1A* | 2.93E-04 |  | *SNCA* | 1.89E-03 |  |  | *SNCA* | 5.54E-04 |  |  |
|  |  | *HADH* | 8.02E-04 |  |  |  |  |  | *ITPR3* | 6.17E-04 |  |  |
|  |  | ***HTR3A*** | 1.17E-03 |  |  |  |  |  | ***HTR1B*** | 9.63E-04 |  |  |
|  |  | ***HTR3B*** | 1.45E-03 |  |  |  |  |  | *KYNU* | 2.20E-03 |  |  |
|  |  | *GRIN2A* | 3.03E-03 |  |  |  |  |  | *MAPK3* | 2.75E-03 |  |  |
|  |  | *INMT* | 3.24E-03 |  |  |  |  |  | *GNAO1* | 2.87E-03 |  |  |
|  |  | ***HTR1B*** | 3.43E-03 |  |  |  |  |  | ***HTR4*** | 5.21E-03 |  |  |
|  |  | *PTGS2* | 3.95E-03 |  |  |  |  |  |  |  |  |  |

In bold, genes belonging to the 5-HT-core gene set. OUD, opioids use disorder; SUD, substance use disorder.

**Supplementary Table 6**. Results of the S-MultiXcan analysis.

|  | DISORDER | Number of computed genes | Number of total significant genes (FDR 5%) | Number of DA significant genes (FDR 5%) | Number of 5-HT significant genes (FDR 5%) |
| --- | --- | --- | --- | --- | --- |
| ADDICTION | **Alcohol Dependence** | 14201 | 0 | 0 | 0 |
|  | **Cocaine Dependence** | 14182 | 0 | 0 | 0 |
|  | **CUD** | 14193 | 2 | 0 | 0 |
|  | **OUD** | 13069 | 212 | 7 | 3 |
|  | **SUD** | 14175 | 339 | 10 | 4 |
| AGGRESSION | **AB** | 14171 | 0 | 0 | 0 |
|  | **ADHD-DBD** | 14164 | 0 | 0 | 0 |
| RELATED BEHAVIOURAL TRAITS | **Anxiety: worrier / anxious feelings** | 14201 | 309 | 5 | 1 |
|  | **Irritability** | 14201 | 160 | 2 | 2 |
|  | **Neuroticism score** | 14201 | 391 | 9 | 2 |
|  | **Risk-taking behaviour** | 14200 | 48 | 0 | 0 |

5-HT, serotonin; AB, antisocial behaviour; ADHD-DBD, attention-deficit and hyperactivity disorder comorbid with disruptive behaviour; CUD, cannabis use disorder; DA, dopamine; OUD, opioids use disorder; SUD, substance use disorder; Risk-Taking; risk-taking behaviour. FDR, false discovery rate

# Supplementary Figures


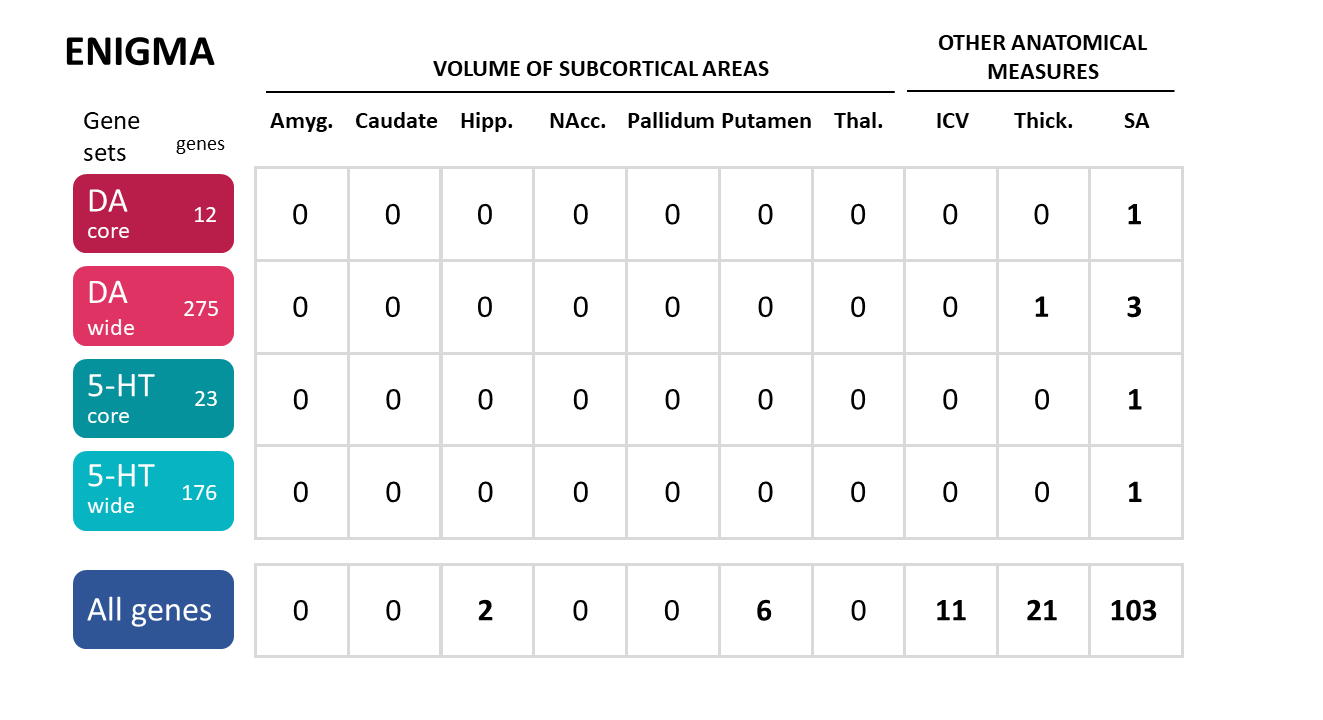


**Supplementary Figure 1.** **Number of significant genes in the gene-based analyses of ENIGMA summary statistics of brain anatomical measures.** Significant genes have overcome a multiple-testing correction of 5% False Discovery Rate, FDR. 5-HT, serotonin; DA, dopamine. Amyg., amygdala volume; Caudate, caudate volume; Hipp., hippocampus volume; ICV, intra-cranial volume; NAcc., nucleus accumbens volume; Pallidum, pallidum volume; Putamen, putamen volume; SA, surface area; Thal., thalamus volume; Thick., cortical thickness.

# References

1. Walters RK, Polimanti R, Johnson EC, McClintick JN, Adams MJ, Adkins AE, Aliev F, Bacanu SA, Batzler A, Bertelsen S, et al. Transancestral GWAS of alcohol dependence reveals common genetic underpinnings with psychiatric disorders. *Nat Neurosci* (2018) 21:1656–1669. doi: 10.1038/s41593-018-0275-1

2. Sherva R, Wang Q, Kranzler H, Zhao H, Koesterer R, Herman A, Farrer LA, Gelernter J. Genome-wide association study of cannabis dependence severity, novel risk variants, and shared genetic risks. *JAMA Psychiatry* (2016) 73:472–480. doi: 10.1001/jamapsychiatry.2016.0036

3. Johnson EC, Demontis D, Thorgeirsson TE, Walters RK, Polimanti R, Hatoum AS, Sanchez-Roige S, Paul SE, Wendt FR, Clarke TK, et al. A large-scale genome-wide association study meta-analysis of cannabis use disorder. *The Lancet Psychiatry* (2020) 7:1032–1045. doi: 10.1016/S2215-0366(20)30339-4

4. Cabana-Domínguez J, Shivalikanjli A, Fernàndez-Castillo N, Cormand B. Genome-wide association meta-analysis of cocaine dependence: Shared genetics with comorbid conditions. *Prog Neuro-Psychopharmacology Biol Psychiatry* (2019) 94:109667. doi: 10.1016/j.pnpbp.2019.109667

5. Polimanti R, Walters RK, Johnson EC, McClintick JN, Adkins AE, Adkins DE, Bacanu SA, Bierut LJ, Bigdeli TB, Brown S, et al. Leveraging genome-wide data to investigate differences between opioid use vs. opioid dependence in 41,176 individuals from the Psychiatric Genomics Consortium. *Mol Psychiatry* (2020) 25:1673–1687. doi: 10.1038/s41380-020-0677-9

6. Deak JD, Zhou H, Galimberti M, Levey DF, Wendt FR, Sanchez-Roige S, Hatoum AS, Johnson EC, Nunez YZ, Demontis D, et al. Genome-wide association study in individuals of European and African ancestry and multi-trait analysis of opioid use disorder identifies 19 independent genome-wide significant risk loci. *Mol Psychiatry* (2022) 27:3970–3979. doi: 10.1038/s41380-022-01709-1

7. Schoeler T, Baldwin J, Allegrini A, Barkhuizen W, McQuillin A, Pirastu N, Kutalik Z, Pingault JB. Novel Biological Insights Into the Common Heritable Liability to Substance Involvement: A Multivariate Genome-wide Association Study. *Biol Psychiatry* (2022) 93:524–535. doi: 10.1016/j.biopsych.2022.07.027

8. Demontis D, Walters RK, Rajagopal VM, Waldman ID, Grove J, Als TD, Dalsgaard S, Ribasas M, Bybjerg-Grauholm J, Bækvad-Hansen M, et al. Risk variants and polygenic architecture of disruptive behavior disorders in the context of attention-deficit/hyperactivity disorder. *Nat Commun* (2021) 12: doi: 10.1038/s41467-020-20443-2

9. Tielbeek JJ, Johansson A, Polderman TJC, Rautiainen MR, Jansen P, Taylor M, Tong X, Lu Q, Burt AS, Tiemeier H, et al. Genome-wide association studies of a broad spectrum of antisocial behavior. *JAMA Psychiatry* (2017) 74:1242–1250. doi: 10.1001/jamapsychiatry.2017.3069

10. Ip HF, van der Laan CM, Krapohl EML, Brikell I, Sánchez-Mora C, Nolte IM, St Pourcain B, Bolhuis K, Palviainen T, Zafarmand H, et al. Genetic association study of childhood aggression across raters, instruments, and age. *Transl Psychiatry* (2021) 11: doi: 10.1038/s41398-021-01480-x

11. Meier SM, Trontti K, Purves KL, Als TD, Grove J, Laine M, Pedersen MG, Bybjerg-Grauholm J, Bækved-Hansen M, Sokolowska E, et al. Genetic Variants Associated with Anxiety and Stress-Related Disorders: A Genome-Wide Association Study and Mouse-Model Study. *JAMA Psychiatry* (2019) 76:924–932. doi: 10.1001/jamapsychiatry.2019.1119

12. Karlsson Linnér R, Biroli P, Kong E, Meddens SFW, Wedow R, Fontana MA, Lebreton M, Tino SP, Abdellaoui A, Hammerschlag AR, et al. Genome-wide association analyses of risk tolerance and risky behaviors in over 1 million individuals identify hundreds of loci and shared genetic influences. *Nat Genet* (2019) 51:245–257. doi: 10.1038/s41588-018-0309-3
